# Kidney Allograft Torsion After Simultaneous Pancreas Kidney Transplantation: Case Report and Review of Literature

**DOI:** 10.1155/crit/2902758

**Published:** 2025-01-19

**Authors:** Ayato Obana, Miho Akabane, Matthew Hamilton, Kejal Shah, Rithin Sai Punjala, Ashley Limkemann, Austin Schenk, Navdeep Singh, Amer Rajab, Ginny Bumgardner, Kenneth Washburn, Musab Alebrahim

**Affiliations:** Comprehensive Transplant Center, Department of Surgery, Ohio State University Wexner Medical Center, Columbus, Ohio, USA

## Abstract

Kidney allograft torsion (KAT) is a rare but critical complication of kidney transplantation that can lead to graft loss due to acute ischemia. This report presents a case of KAT resulting in graft loss 9 months following intraperitoneal simultaneous pancreas and kidney (SPK) transplant and reviews previous reports to identify potential high-risk features. A 38-year-old female with end-stage renal disease secondary to Type 1 diabetes mellitus underwent an intraperitoneal enteric drained SPK transplant. Nine months post-transplantation, she presented with nausea, vomiting, severe abdominal pain, decreased urine output, and diarrhea. An ultrasound showed moderate hydronephrosis and no blood flow to the renal hilum. Exploratory laparotomy revealed a necrotic renal allograft twisted 360° counterclockwise on its vascular pedicles. Despite detorsion, the graft showed no signs of viability, necessitating transplant nephrectomy. This case highlights the rarity and severity of KAT, particularly in intraperitoneal kidney transplants. The patient's low body mass index (BMI) (23.4 kg/m^2^), female sex (wider pelvis), and minimal intra-abdominal adhesions may have contributed to increased graft mobility, predisposing to KAT. Other potential risk factors include elongated vascular pedicle and immunosuppression-related reduced adhesion formation. The nonspecific presentation of KAT emphasizes the need for high clinical suspicion and prompt ultrasonographic evaluation in cases of graft abnormalities. This report underscores the importance of considering patient- and graft-specific factors in assessing KAT risk and the critical nature of early detection and intervention to prevent graft loss.

## 1. Introduction

Kidney allograft torsion (KAT) is a rare but critical complication of kidney transplantation (KT) that can result in graft loss due to acute ischemia if not promptly addressed [1–6]. Rapid diagnosis followed by surgical detorsion and nephropexy is essential for preserving allograft function [1–6]. Previous studies have shown that KAT, while more prevalent in intraperitoneal transplants due to increased graft mobility, often eludes prompt diagnosis. This diagnostic delay frequently stems from KAT's nonspecific clinical manifestations—including pyrexia, abdominal discomfort, deteriorating renal function, oliguria, gastrointestinal disturbances, and edema—which closely mimic more common post-transplant complications such as allograft rejection or ureteral obstruction [7]. Consequently, the diagnosis may be obscured even when timely imaging studies are performed [8].

While early laparotomy or prophylactic measures are warranted to prevent graft loss, patient- and donor-specific high-risk features for KAT have not been thoroughly explored in the literature. This study presents a case of KAT that resulted in graft loss following an intraperitoneal simultaneous pancreas and kidney (SPK) transplant and reviews previous reports to identify potential high-risk features associated with KAT.

## 2. Case Presentation

A 38-year-old female (height 162.2 cm, weight 61.6 kg, and body mass index (BMI) 23.4 kg/m^2^) with end-stage renal disease secondary to Type 1 diabetes mellitus (T1DM) diagnosed at the age of 20 underwent intraperitoneal enteric drained SPK transplantation with ureteral stent placement on December 23, 2023. Ureteral stent placement is our center's standard protocol to prevent urological complications and ensure systematic removal at 3–4 weeks post-transplantation. The kidney allograft was a right kidney from a brain-dead donor. The vena cava was used to create an extension conduit of the donor's right vein. The arterial anatomy of the allograft included two renal arteries on a common aortic cuff, each measuring 6 cm in length and separated by a 1 cm interval. The allograft also had a solitary ureter.

Following the midline incision, a pancreas transplant was performed first on the right side. The kidney was implanted in the left lower intraperitoneal space with standard end-to-side anastomoses of the donor artery and vein to the recipient's external iliac artery and vein. The recipient tolerated the procedure well, with an uneventful postoperative course.

Antithymocyte globulin (ATG) (3.68 mg/kg total) and a rapid prednisone taper were used for induction. Mycophenolate mofetil and tacrolimus were used for maintenance immunosuppression. During her inpatient stay, her urine output (UOP) averaged 3.3 L/day, and creatinine was 0.58 mg/dL on the day of discharge (Postoperative Day (POD) 4).

On January 9, 2024, this recipient was readmitted with a creatinine level of 1.7 mg/dL, and a renal biopsy confirmed acute cellular rejection (Banff 1–2B). She was treated with 500 mg of IV SoluMEDROL for 3 days and an additional 1.5 mg/kg of ATG. After 5 days of hospitalization, her creatinine improved to 1.1 mg/dL, and she was discharged.

**Figure 1:**
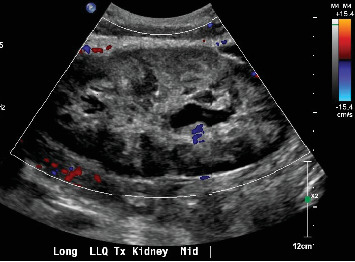
Transverse ultrasonographic image of a renal allograft. Despite the application of appropriate low-flow Doppler settings, no detectable blood flow was observed within the renal graft parenchyma or the principal renal vasculature, suggestive of graft thrombosis or severe ischemia.

On September 23, 2024, she presented with nausea/vomiting, severe abdominal pain, significantly decreased UOP, and diarrhea that had started 4 days prior. She reported feeling well until these symptoms began, with the pain worsening after eating. Laboratory results showed a serum creatinine of 4.5 mg/dL and a white blood cell count of 20,000/*μ*L. Renal ultrasound (US) revealed moderate hydronephrosis, heterogeneous renal parenchyma, and no blood flow to the renal hilum and arcuate vessels (Figure 1).

**Figure 2:**
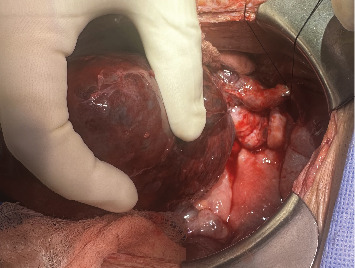
Intraoperative photographs demonstrating renal allograft torsion. Surgical exploration revealed significant congestion of the transplanted kidney, indicative of compromised vascular flow secondary to graft rotation.

The patient was urgently taken to the operating room for exploratory laparotomy. Upon reopening the prior midline incision, a moderate volume of sanguinous ascites was encountered in the abdominal cavity. Contrary to expectations, there were remarkably few adhesions in the intraperitoneal space. The renal allograft appeared necrotic, displaying significant engorgement and parenchymal hemorrhage (Figure 2). The graft was found to have twisted almost 360° counterclockwise on its vascular pedicles, and the ureter was running around the twisted vascular pedicle. Detorsion of the allograft was performed, followed by restoration of the ureter to its proper anatomical position. Notably, despite successful correction of the torsion, the graft persistently exhibited signs of severe engorgement and failed to regain normal parenchymal color. Given these concerning intraoperative observations and the allograft's failure to exhibit signs of viability postdetorsion, the surgical team made the clinical decision to proceed with transplant nephrectomy. Concomitantly, a hemodialysis (HD) catheter was placed intraoperatively to facilitate postsurgical renal replacement therapy. The patient subsequently underwent intermittent postoperatively. The postoperative course was uneventful, and she was discharged POD 7.

The final pathological examination of the allograft revealed extensive renal infarction involving nearly the entire organ. The specimen measured 14.0 × 9.7 × 6.0 cm. Gross examination demonstrated diffuse congestion and hemorrhage throughout the renal parenchyma. A thrombus measuring 3.9 × 1.4 × 1.0 cm was identified within the renal vein, completely obstructing the lumen.

## 3. Discussion

This case report provides a detailed account of KAT, an exceedingly rare but critical complication that led to graft loss. What makes this case particularly unusual is the timing—occurring 9 months post-transplant—and the absence of significant intra-abdominal adhesions during reoperation.

KAT, while exceedingly rare, carries significant clinical implications. To date, 27 cases of KAT have been reported in the literature, with nine cases resulting in nonviable grafts necessitating transplant nephrectomy (Table 1). KAT predominantly manifests in the early postoperative period; however, cases have been documented up to 10 years post-transplantation [9]. Of the 27 reported cases, 23 (85%) involved intraperitoneal KT, likely due to the increased mobility of intraperitoneal grafts, which heightens the risk of torsion. Notably, all cases requiring transplant nephrectomy were intraperitoneal placements. In contrast, extraperitoneal cases exhibited limited graft mobility, potentially resulting in less severe torsion, some preservation of blood flow, and a higher likelihood of graft salvage. In the present case, the patient presented with intermittent abdominal pain for 4 days. An immediate US on admission revealed nearly absent blood flow to the graft. The vascular pedicle had rotated 360°, likely causing venous occlusion, which led to graft enlargement and edema. Given the 4-day duration of symptoms, irreversible changes had likely occurred, rendering graft salvage impossible. Common factors among cases resulting in graft loss include intraperitoneal graft placement in all cases, a delay of over 24 h between symptom onset and surgical intervention [3, 4], and complete cessation of blood flow due to torsion [1, 2, 5, 6, 10].

The clinical presentation of KAT is characterized by a constellation of nonspecific symptoms, including abdominal pain, nausea, vomiting, oliguria, and elevated serum creatinine levels, making it difficult to distinguish from more common causes of graft dysfunction such as rejection, urinary tract infection, or obstruction. In our case, pathological examination revealed a significant thrombus (3.9 × 1.4 × 1.0 cm) completely obstructing the renal vein lumen. While renal vein thrombosis (RVT) was observed in our case, it likely developed secondary to torsion-induced venous stasis rather than as a primary event. This sequence highlights an important pathophysiological cascade: the 360° torsion led to venous occlusion, resulting in congestion and subsequent thrombus formation. RVT, occurring in 0.1%–4.2% of renal transplant cases [11], typically presents as acute graft dysfunction in the immediate post-transplant period when it occurs as a primary event [12]. However, in cases of KAT, the development of RVT represents a downstream consequence of prolonged vascular compromise, further complicating any potential for graft salvage. This underscores the critical importance of early recognition and intervention in cases of suspected KAT, as the window for preventing irreversible vascular complications may be limited. Therefore, clinicians should maintain a high index of suspicion for KAT, particularly in cases of intraperitoneal KT, such as in SPK transplantation. Patient education is also crucial; recipients should be instructed to seek immediate medical attention if they experience abdominal pain or decreased UOP. Early US evaluation for blood flow may reduce the incidence of graft loss.

In the SPK transplantation protocol at our center, the kidney allograft is placed in the extraperitoneal space through a midline incision to reduce KAT risk [13] and shield it from pancreatic inflammation. While intraperitoneal kidney placement provides excellent surgical exposure, it carries increased risks of graft mobility and torsion [14]. Extraperitoneal placement, as practiced at our center and others, offers more stable positioning and easier access for biopsies, although surgical exposure can be more limited [15]. When intraperitoneal placement is required, it is reported that pexing the lateral peritoneal reflection adjacent to the colon over the transplanted kidney may help stabilize the graft. While right-sided placement is typically preferred due to favorable iliac vessel anatomy for vascular anastomoses, in cases where left-sided placement is performed, the sigmoid colon and its mesentery can provide additional coverage of the allograft [16].

Pelvic anatomy appears to play a role in KAT risk, particularly regarding gender differences. The characteristically wider female pelvis provides more intraperitoneal space [17], potentially allowing greater graft mobility. This anatomical feature may help explain why females represent nearly half (13/28) of reported KAT cases despite men having higher rates of KT overall [18]. The combination of wider pelvic dimensions and other patient-specific factors, such as BMI, may be associated with KAT risk in female recipients.

In the present case, the patient's low BMI (23.4 kg/m^2^) likely contributed to KAT. Low BMI is associated with reduced intra-abdominal fat and minimal adhesions, increasing graft mobility and predisposing the patient to torsion. In support of this, 17 (63%) out of the 27 reported cases involved patients with long-standing insulin-dependent T1DM, and 18 cases (67%) occurred in SPK recipients, both groups known to have lower BMIs [19, 20]. Additionally, it is reported that high BMI is associated with high intra-abdominal pressure, suggesting that low BMI patients may have decreased intra-abdominal pressure, potentially allowing greater graft mobility [21]. However, despite this potential connection, only three of the 27 previously reported cases of KAT included BMI information, suggesting that the link between BMI and KAT has not been sufficiently explored [22]. To further clarify the risk factors associated with KAT, future case reports should include detailed patient data, including BMI.

Graft-related factors potentially contributing to KAT include small graft size, elongated vascular pedicle, and lack of adhesion formation. In the present case, the graft length was average size, measuring 12 cm in length. Notably, a previous case of KAT occurred in an extraperitoneal transplant—a setting typically considered lower risk for torsion—where the graft size was relatively small, at 11 cm [23]. Moreover, excessive length of the vascular pedicle or ureter has been identified as a potential etiological factor for this condition [2, 24, 25]. Therefore, the left kidney graft is more likely to have KAT compared to the right kidney graft unless a vascular extension graft is used. These anatomical characteristics, both from the recipient and the graft, may increase graft mobility, elevating the risk of torsion.

Another proposed mechanism for KAT involves diminished adhesion formation, which is common in immunosuppressed recipients [26]. The standard immunosuppressive regimen in our case, including tacrolimus and mycophenolate mofetil, may have contributed to reduced adhesion formation through distinct mechanisms: tacrolimus by decreasing TGF-beta levels and preventing rejection-related adhesions [27] and mycophenolate mofetil by inhibiting adhesion molecule expression such as ICAM-1 and VCAM-1 [28]. While this reduction in adhesion formation is beneficial for preventing rejection, it may have contributed to increased graft mobility in this case. Sirolimus, a commonly used immunosuppressant with antifibrotic properties, has been associated with an increased incidence of wound-related complications, suggesting that the immunosuppressive regimen may create an adhesion-deficient environment, potentially allowing unrestricted rotation of the renal allograft and increasing the risk of torsion [29].

KAT represents a rare but serious complication that frequently leads to graft loss and poses significant diagnostic challenges. Evidence suggests that recipients with low BMI may be at an elevated risk for developing KAT. Furthermore, the risk may be due to factors such as small graft size or elongated vascular pedicles. In the post-KT period, clinicians should maintain a high index of suspicion for KAT when graft abnormalities are observed. Prompt ultrasonographic evaluation is imperative in such cases to assess the possibility of torsion and mitigate the risk of graft loss. Future research should focus on developing predictive models for high-risk patients and investigating optimal imaging protocols for early detection. Prospective studies evaluating surgical techniques for graft fixation and the impact of different immunosuppressive regimens on KAT risk could help establish evidence-based preventive strategies.

## Figures and Tables

**Table 1:** Summary of case reports of kidney allograft torsion.

**Case**		**Sex**	**Age**	**BMI**	**Position**	**Tx kidney**	**KP or K**	**Donor**	**Interval to KAT**	**Preop dx**	**Intervention**	**GS after KAT**	**Primary disease**
Abbitt et al.	1990	M	2.5 years	NA	Intraperitoneal	NA	K	Living	8 weeks	Yes	TN	9 weeks	Prune belly

Marvin, Halff, and Elshihabi	1995	M	26 months	NA	Intraperitoneal	NA	K	Deceased	6 weeks	Yes	TN	6 weeks	Prune belly

West et al.	1998	M	57 years	NA	Intraperitoneal	L	SPK	Deceased	3 weeks	Yes	Detorsion	NA	44 years insulin-dependent T1DM
		M	26 years	NA	Intraperitoneal	L	SPK	Deceased	22 months	No	Detorsion/nephropexy	NA	25 years insulin-dependent T1DM
		F	41 years	NA	Intraperitoneal	L	SPK	Deceased	5 months	No	TN	5 months	26 years insulin-dependent T1DM
		F	46 years	NA	Intraperitoneal	L	SPK	Deceased	21 months	Yes	Detorsion/nephropexy	NA	22 years insulin-dependent T1DM

Roza, Johnson, and Adams	1998	M	45 years	NA	Intraperitoneal	L	SPK	NA	16 months	No	TN	16 months	36 years insulin-dependent T1DM
		F	39 years	NA	Intraperitoneal	L	SPK	NA	11 months	No	TN	11 months	28 years insulin-dependent T1DM

Badet	2003	F	31 years	NA	Intraperitoneal	NA	SPK	NA	7 months	NA	Detorsion	NA	NA

Rodrigues, Hering, and Gil	2008	M	52 years	NA	Intraperitoneal	R	SPK	Deceased	NA	No	TN	120 days	Long-standing T1DM

Nangia and Saad	2009	F	48 years	NA	Intraperitoneal	NA	SPK	NA	10 years	No	Detorsion/nephropexy	NA	T1DM

Lucewicz et al.	2011	M	27 years	NA	Intraperitoneal	L	K	Living	10 weeks	No	TN	10 weeks	Chronic interstitial nephritis

Kaynar	2013	M	55 years	NA	Intraperitoneal	L?	K	Living	2 years	Yes	Detorsion/nephropexy	NA	Both native nephrectomies for polycystic disease

Ozmen et al.	2013	M	44 years	NA	Extraperitoneal	L	K	Living	4 days	Yes	Detorsion/nephropexy	NA	Mesangioproliferative glomerulonephritis, length gap between A&V

Winter	2013	M	68 years	NA	Extraperitoneal	NA	K	Deceased	4 h	No	Detorsion	NA	Small kidney

Sosin	2014	M	42 years	NA	Extraperitoneal	NA	K	Deceased	1 day	No	Detorsion/nephropexy	NA	Glomerulonephritis 2nd KTx

Sofue et al.	2015	M	34 years	NA	Intraperitoneal	NA	SPK	Deceased	3 months	Yes	Detorsion/nephropexy	NA	T1DM
		M	37 years	NA	Intraperitoneal	NA	SPK	Deceased	1 month	Yes	Detorsion/nephropexy	NA	T1DM

Dewan et al.	2016	F	60 years	26.6	Intraperitoneal	NA	SPK	Deceased	2 years	No	Detorsion/nephropexy	NA	T1DM

Serrano et al.	2017	F	49 years	NA	Intraperitoneal	NA	SPK	Living (K), Deceased (P)	3 years	Yes	Detorsion/nephropexy	8 years	T1DM

Narasimhan	2017	F	34 years	NA	Intraperitoneal	NA	SPK	Deceased	5 weeks	Yes	Detorsion/nephropexy	NA	T1DM, her symptoms began following her first postoperative sexual intercourse

Torabi	2018	F	28 years	NA	Intraperitoneal	L	SPK	Deceased	7 months	No	Detorsion/nephropexy	NA	T1DM

Greco, Mulligan, and Yoo	2020	F	69 years	25.9	Extraperitoneal	R	K	Deceased	1 day	No	Detorsion/nephropexy	NA	ADPKD, small graft 11 cm

Vincenzi et al.	2021	F	38 years	NA	Intraperitoneal	NA	SPK	Deceased	7 months	Yes	Detorsion/nephropexy	NA	28 years insulin-dependent T1DM, nephropexy failed

Wendy	2021	M	7 years	NA	Intraperitoneal	L	K	Living	3 years	Yes	TN	NA	Renal dysplasia, S/p b/l nephrectomy

Tan	2022	M	39 years	NA	Intraperitoneal	L	SPK	Deceased	3 months	Yes	Detorsion/nephropexy	NA	T1DM

Obana	2024	F	38 years	23.6	Intraperitoneal	R	SPK	Deceased	9 months	No	TN	NA	T1DM insulin-dependent 20 years

Abbreviations: A&V: artery and vein, ADPKD: autosomal dominant polycystic kidney disease, b/l: bilateral, GS: graft survival, h: hour, intraop: intraoperative, K: kidney, KAT: kidney allograft torsion, KTx: kidney transplant, L: left, NA: not applicable, P: pancreas, preop: preoperative, R: right, s/p: status post, T1DM: Type 1 diabetes mellitus, TN: transplant nephrectomy.

## Data Availability

The datasets generated and analyzed during the current study are available from the corresponding author upon reasonable request.
